# Investigating informed choice in screening programmes: a mixed methods analysis

**DOI:** 10.1186/s12889-022-14685-6

**Published:** 2022-12-12

**Authors:** Natalie Tyldesley-Marshall, Amy Grove, Iman Ghosh, Laura Kudrna, Abimbola Ayorinde, Megha Singh, Edward Mehaan, Aileen Clarke, Sian Taylor-Phillips, Lena Al-Khudairy

**Affiliations:** 1 Applied Research Collaboration West Midlands, Warwick Medical School, University of Warwick, CV4 7AL Coventry, UK; 2 Public Health and Health Technology Assessment and Implementation Science, Warwick Medical School, University of Warwick, CV4 7AL Coventry, UK; 3 Present address : Institute of Applied Health Research, University of Birmingham, B15 2TT Edgbaston, UK; 4 Monash University, Wellington Road, VIC 3800 Clayton, Melbourne, Australia

**Keywords:** Screening, Fetal anomalies, Cancer, Public policy, Informed choice, Mixed methods, Decision-making, Decision aids, Health communication, Non-invasive pregnancy testing

## Abstract

**Background:**

Screening programmes aim to identify individuals at higher risk of developing a disease or condition. While globally, there is agreement that people who attend screening should be fully informed, there is no consensus about how this should be achieved. We conducted a mixed methods study across eight different countries to understand how countries address informed choice across two screening programmes: breast cancer and fetal trisomy anomaly screening.

**Methods:**

Fourteen senior level employees from organisations who produce and deliver decision aids to assist informed choice were interviewed, and their decision aids (*n* = 15) were evaluated using documentary analysis.

**Results:**

We discovered that attempts to achieve informed choice via decision aids generate two key tensions (i) between improving informed choice and increasing uptake and (ii) between improving informed choice and comprehensibility of the information presented. Comprehensibility is fundamentally at tension with an aim of being fully informed. These tensions emerged in both the interviews and documentary analysis.

**Conclusion:**

We conclude that organisations need to decide whether their overarching aim is ensuring high levels of uptake or maximising informed choice to participate in screening programmes. Consideration must then be given to all levels of development and distribution of information produced to reflect each organisation’s aim. The comprehensibility of the DA must also be considered, as this may be reduced when informed choice is prioritised.

**Supplementary Information:**

The online version contains supplementary material available at 10.1186/s12889-022-14685-6.

## Introduction

The aim of health screening is to identify asymptomatic individuals at higher risk of developing a particular disease or condition [1] in order to offer care or treatment to improve health and wellbeing. Across the globe, screening programmes aim to maximise uptake of screening to benefit population health [2], as well as to ensure cost-effectiveness of programmes [2, 3]. However, programmes which put too great an emphasis on uptake and efficiency of screening programmes run the risk that people who are invited for screening are not supported to make an informed choice about their participation [2].

Informed choice has been defined in international literature as a choice that is consistent with an individual’s values and which is based on adequate information [2, 4, 5]. Adequate information is thought to include risks, benefits, limitations and uncertainties of undertaking screening or not, and information on the condition itself, the screening process, and subsequent decisions that screening may lead to [6, 7]. People invited to screening should also be given the opportunity to reflect on the potential consequences of the screening and receive sufficient support to enable them to make the right choice for them, given their values and circumstances [6]. Undesired and potentially harmful outcomes include the screening finding that the person has a condition when they do not, or failing to detect a condition (known as false positives and false negatives respectively). False positives may lead to unnecessary treatment, or “overdiagnosis”, where a condition is diagnosed “*that would otherwise not cause symptoms or harm to a patient during his or her lifetime*” for which the person may then receive treatment, or “overtreatment” [8]. However, there are concerns that including information on these outcomes in order to facilitate informed choice may have a negative impact on screening uptake [3].

### Strategies for improving informed choice in the context of breast cancer, or fetal trisomy anomaly, screening 

To support informed choice, screening programmes across the world have developed interventions to aid people making decisions about screening, which may be self-administered or administered by healthcare professionals (HCPs) [5]. These are sometimes called decision aids (DAs) [9]. DAs provide summary information written in simple, non-technical language, which are intended to be used during healthcare consultations or made available for a person to take away. DAs can come in a range of formats, such as digital or hard copy leaflets and webpages, and many are designed to be used in conjunction with consultations with HCPs [10]. They seek to improve knowledge regarding screening options and consequences to assist people to reach the right decision for them [11], simplifying complex discussions [12]. Evidence has shown that DAs used with HCPs are more effective in increasing understanding and recall of medical facts than HCPs alone [13]. People have also been shown to arrive at a decision earlier in the process when using a DA [14]. Most countries rely on individuals to make their own decisions in relation to whether or not to be screened, and screening programmes provide DAs to help people in these choices rather than making screening mandates.

Health behaviour models can be useful in considering how individuals decide whether or not to attend screening. The most frequently used theories to explain health behaviour are the health belief model, the trans-theoretical model, theory of planned behaviour, and social cognitive theory, although these focus on different aspects of behaviour [15, 16]. The additional theory of care seeking behaviour was developed specifically to explore the factors affecting women’s decisions to undergo mammography screening or not [16]. Collectively, these models highlight the importance of cues to action, and the individual’s perception of how severe the condition is, how susceptible they are to it (risk factors), the costs, benefits, barriers, and outcomes to screening, the feasibility and efficacy of screening, their ability to undertake the action, their intention to be screened, and what others do and would approve of [15–17]. However, while DAs are “underpinned by theories on how we make decisions under risk and uncertainty, and the factors influencing our judgement and choices […] it is reported that theories are underused” [11] (p.3105).

While there is consensus between HCPs, researchers, and policymakers that informed choice should be prioritised as an important component of screening programme development and delivery, consensus has not been reached about *how* this should be achieved, or systematically measured [18]. We recently produced a rapid systematic review which found that DAs *are* effective in facilitating informed choice for screening within the context of breast cancer screening and fetal trisomy anomaly screening. We found that after using DAs, knowledge was improved on the screening procedure, the benefits and harms of screening, and the conditions being screened for, and women were more likely to make an informed choice for breast cancer screening [19, 20], and prenatal screening [21, 22]. (Effective DAs were found online and ‘offline’, and ranged from a physical leaflet, an interactive online tool, to a face-to-face course (Ayorinde et al., forthcoming).

In this study we were commissioned by the funder to explore how organisations involved in the production and delivery of screening programmes across eight countries address informed choice. In addition to the systematic review mentioned previously, we interviewed senior staff members from each organisation and assessed the DAs that their organisation provides to people invited to screening. We aimed to explore how informed choice is enacted through the content and distribution of these materials, and we contribute an exploration of informed choice by examining the tensions between informed choice and uptake of screening.

## Methods

### Selection of participants and materials

This study used mixed methods with participation across different countries. During our preliminary scoping of the literature and in negotiation with the research funder and clinical experts, the research team selected fetal trisomy anomaly screening and breast cancer screening. We recognised the delivery and support for screening and diagnosis differs by programme, so selected two different programmes to explore contrasts (and potential similarities) between these. We aimed to explore how organisations involved in the production and delivery of screening programmes address informed choice and planned to invite different participants (potentially from the same organisation) to interview. Screening programmes for similar conditions may have reduced the scope of participants who could contribute to our study.

We compiled a list of developed countries that had organised population-based screening programmes, so in this sense, were comparable to the United Kingdom (UK). Participants were senior-level employees working in public health, or in other organisations responsible for developing information materials for people deciding whether to be screened for fetal trisomy anomaly or breast cancer. Potential participants were identified through contacting relevant national or local organisations who provide screening information for each country, or through existing contact networks. We requested that interviews would be undertaken in English. From those that responded to our efforts at contact, none declined to take part in the interviews; some referring us to more suitable contacts. This snowball sampling approach led us to find that often, one organisation is responsible for screening, as is the case with the UK National Screening Committee (UK NSC), and requests within that organisation tended to generate contacts to one or two people: one for breast cancer screening, and another for fetal trisomy anomaly screening.

Given time and resource constraints of the project, once participants from eight countries agreed to take part - Norway, Denmark, Sweden, Netherlands, Australia, New Zealand, Canada, and England - recruitment was stopped.

All potential participants were sent an email describing the research in brief, asking if they would be interested and if they had any further questions. A link to a project information sheet and consent form was attached to subsequent emails for their consideration.

### Data collection

#### Semi-structured interviews

Semi-structured interviews were used with participants to explore their perspectives of informed choice in screening. The semi-structured approach enabled the order and wording of questions to be adapted during the interview for better understanding, yet allowing questions to be similar enough for responses to be compared across interviews [23]. An interview schedule (Additional file 1) was developed to guide questioning during the interviews. This was developed iteratively by the research team in discussions with the funder (UK NSC) and key studies identified during the study’s preliminary scoping of the literature.

Interviews were undertaken by experienced and trained researchers (IG, MS, or NT) from April to July 2021. Informed consent was re-established at the start of each interview. Initial interviews were observed online by a senior researcher (LK) to ensure quality of the interview, offer points of clarification, and suggest further probing where required. Field notes of researcher reflections and initial thoughts regarding data were written up in field journals immediately after each interview and shared with the data collection team.

Each participant was interviewed once, and interviews lasted approximately 35–65 min. Interviews were conducted and recorded using Microsoft Office Teams (Version 1.0) [24]. All interviews were video- and audio-recorded, unless poor internet connection limited video recording. All participants were interviewed during their working hours; however, most were working remotely during the pandemic restrictions. To the best of our knowledge, all participants were alone.

#### Documentary analysis

During or following the interview, the research team requested DAs for both fetal trisomy anomaly and breast cancer screening if available. This sometimes included signposting to publicly accessible websites to find screening information, or digital copies of DAs which were sent to the research team. If information materials were not provided by participants, or they were not certain where these could be located, members of the research team (IG, LAK) searched for and accessed materials from organisational websites. (In the case of Denmark, the DA was not in English, and so was translated using Google Translate [25]).

DAs produced by the participants’ organisations were collected to be assessed against a validated checklist to assess the quality of information provided. An updated version of the 2005 International Patient Decision Aid Standards (IPDAS) Quality Criteria Checklist for Patient Decision Aids [26] was used [13]. LAK and IG independently evaluated the content of the DAs across all eight domains (including sub-domains (statement of aims, screening options, screening outcomes, information accuracy, decision making support, conflict of interest, layout, and reliability of information) using the IPDAS / Picker Institute scoring system [13] (see Additional file 2).

### Characteristics of participants and materials

#### Semi-structured interviews

Fourteen participants were interviewed, recruited from eight countries. In the case of New Zealand, one person was interviewed for both conditions as they were the most appropriate person for both breast and fetal screening, and both conditions were covered in a single interview. In the case of Australia, there was difficulty identifying a participant for fetal trisomy anomaly screening within the timeframe of the research. The characteristics of participants interviewed are reported in Table 1.

**Table 1:** Participant characteristics

Participants (*n* = 14)	
**Gender**	
Female	13
Male	1
**Screening programme/s**	
Breast	7
Fetal	6
Breast and fetal	1
**Country of screening programme**	
Australia	1
Canada	2
Denmark	2
England	2
Netherlands	2
New Zealand	1
Norway	2
Sweden	2
**Job title**	
Leader of screening programme	3
Clinical director of screening programme	2
Manager of screening programme	2
Leader / manager of organisation providing preventative healthcare guidelines	3
Chair of relevant information network	1
Leader / manager of relevant working group of healthcare professionals	2
Advisor for screening programme	1

#### Documentary analysis

Fifteen DAs were assessed from eight countries. Breast cancer screening DAs were almost all leaflets (*n* = 7), while Sweden provided a webpage (Table 2). For breast cancer screening, the length of the DA varied from two to 20 pages. For fetal trisomy anomaly screening, four countries provided a leaflet and three countries provided a webpage; their length ranging from four to 23 pages, and webpages from four to 12 tabs.

**Table 2:** Description of materials

Country	Breast cancer screening (*n* = 8)			Fetal trisomy anomaly screening (*n* = 7)		
	Leaflet	Webpage	Length	Leaflet	Webpage	Length
Australia	√		20 pages			
Canada	√		3 pages		√	5 tabs
Denmark	√		8 pages	√		8 pages
England	√		16 pages	√		4 pages
Netherlands	√		16 pages	√		23 pages
New Zealand	√		6 pages	√		8 pages
Norway	√		2 pages		√	7 tabs
Sweden		√	2 tabs		√	12 tabs

### Data analysis

#### Semi-structured interviews

All recordings of the interviews were professionally transcribed by a company external to the research team. Interview data were analysed thematically, using a qualitative approach, without a pre-specified theoretical perspective [27]. The principles of the Framework Method were followed [28]. Transcripts were checked against recordings to ensure that they were accurate. The transcripts were read to gain a thorough understanding of participant responses, and were coded inductively line-by-line in NVivo 12 Pro [25] by one member of the research team (NT). Emails and supplementary documents sent by the participants following their interviews were also read in detail and coded where relevant. To ensure trustworthiness of the coding, early transcripts were independently coded by a second researcher (AG) and codes were agreed and shared with the larger research team.

Codes were grouped into broader categories [30]. As the analysis progressed, the analytical framework was further refined with subsequent interviews, then cross-checked with researchers who conducted the interviews (IG and LK).

Interpretation of the data and searches for patterns in participant responses were undertaken throughout [28]. The framework matrix was created in a Microsoft Excel 365 (2021) spreadsheet [31], with a row for each interview and a column for each code, and a separate sheet for each broader category. Where an interview contained content labelled for a code, this was summarised and added to the corresponding cell [28].

Both types of screening programme data were entered into one framework matrix. However, these were often separated by type, and colour coding used, to aid searching for similarities and differences between the two types of screening programme, considering the condition-specific differences we anticipated would arise. Fetal trisomy anomaly screening, for example, is time-dependent on the week of gestation; while in breast cancer screening, women within a set age range are identified and contacted by email or letter. Four themes were identified during the initial thematic analysis of the interview data: (1) Development process; (2) Content and Purpose; (3) Delivery and access of screening information and programme; and (4) Wider influences on information and screening.

Our approach to analysis to this point was inductive. However, we set out to reflect and build on these themes within a broader context of the informed choice decision-making literature [14, 12, 7], our systematic review of the effectiveness of DAs for informed choice, and a one-day consensus principles workshop with international representation which we hosted (7 July 2021). In the workshop we presented the preliminary findings of our analysis to research funders, patients, and interview participants that wished to attend. We sought feedback on their views regarding the initial findings, cross-checked our interpretations, and discussed areas for future exploration. This abductive process [32] moved our analysis further and led to the generation of two final themes, which are described in the Results.

#### Documentary analysis

Two researchers (LAK, IG) assessed materials independently. For each DA, each sub-domain from the Picker Institute IPDAS checklist [13] was rated on a scale that ranged from one to five points. A score of one was awarded if the material did not meet the sub-domain statement. Scores of two, three and four were awarded if the material partially met the sub-domain statement. A total score of five was awarded if the material completely fulfilled the sub-domain statement. If the two researchers had different scores, then evidence for each score was compared and discussed, and their new scores, and evidence, recorded in a spreadsheet [31].

## Results

Thematic results are presented below. Results for fetal trisomy anomaly screening and breast screening are presented together, unless there are differences between the two, which are highlighted.

### Increasing informed choice or increasing uptake?

The first theme involved the tension between improving informed choice or increasing uptake. When asked directly about the main purpose of their DA, all but one participant gave a response around informed choice such as fully understanding the benefits and harms of screening. Many participants explicitly stated that it was more important that women be fully informed about screening than whether they participated in screening, and that there were no targets for uptake of screening for their organisation. Many participants from the fetal screening programmes also made statements that the aim of the DA was to enable women to make choices in line with their values and preferences.

“[The core aims of the ‘DA’ are] that women can make a, an informed choice.” Interview 11, Line 592, Informed choice priority for DA

“So it’s not necessarily about driving women to consent and then participate, it’s more about ensuring that whether they choose to participate or not, it’s an intelligent decision.” Interview 2, Line 298, Informed choice over increased uptake priority for DA

“What is the aim [of the DA]? For me, obviously, to be that the person are making decisions that are, related and stands by their values, needs, et cetera.” Interview 10, Line 755, Values and preferences priority for DA

Two participants expressed contrasting sentiments. (Although one of these also stated that informed choice was the priority).

“You should go [...] So, the purpose of the leaflet, as you said, is that you should come. The purpose is to increase the participation.” Interview 4, Line 468, Increased uptake over informed choice priority for DA

“I find this a really complex area because, you know, you’re wanting women to screen. Yet, this is information that they should have, and but it needs to be put in a way that doesn’t put people off. [...] So, in terms of our screening programmes, we recommend breast screening.” Interview 11, Line 624, Increased uptake over informed choice priority for DA

A few expressed the view that the DA was *balancing* the competing aims of increasing informed choice and increasing uptake. Some participants expressed views that their DA contained a clear recommendation for women to be screened while a few other participants believed that their DA did not recommend screening strongly enough. These views all emerged from the breast cancer screening programmes.

“Neither’s [promoting uptake or informed choice] prioritised. I think at the moment it [the DA] is very balanced.” Interview 6, Line 783, Balanced uptake and informed choice

“We do state that then it is a recommendation from the government, or from them... not from the government but there is the guidelines, the [country’s] and the EU guidelines recommend that... that women [between certain ages] participate in breast cancer screening. But that there are benefits and harms and we encourage them to read the information to make up their own mind.” Interview 12, Line 707, Balanced uptake and informed choice

“We’re not pretending it [overdiagnosis] doesn’t happen, but we’re, advising that on the best evidence available, the benefits still outweigh the harms.” Interview 2, Line 372, Recommendation to screen

“I personally think that it [the DA] perhaps, swung too negatively towards the risks of, of breast cancer [screening]. I think a lot of services felt the same at the time.” Interview 6, Line 550, DA does not recommend screening enough

When asked what was the most important information that their DA provided, almost all reported the benefits and harms. A few suggested that the information that allowed people to make the *correct choice* in line with their values and preferences was the most important (in addition to the benefits and harms). A few also reported that the most important information gave the understanding of what the test was and what it involved.

“We mention that there are advantages and disadvantages. Of course it’s important because some people don’t think about disadvantages at all.” Interview 8, Line 348, Most important information is benefits and harms

“[The main aims of the DA are] describing the process of the screening, so… about coming to the unit, that they have to, undress and that their breast will be made—the mammogram will be made, how the reading is… that the reading is done by a radiologist, how the… result is communicated.” Interview 8, Line 319, Most important information is understanding of the test

“I think women have to know what they choose, what their choice is, what consequences of different results are. And I think they have to have information which should allow them to think about what does this information mean about my personal values of life?” Interview 13, Line 433, Most important information is benefits and harms / allows choice in line with values and preferences

However, more in line with an aim of maximising participation, one participant indirectly suggested that the most important information was the recommendation to be screened. (This was for a breast screening programme.)

“Main aim [of the DA] is to... for... fulfilled the [external] demands on talking about the, the benefits and, and the downsides of screening, but it’s a very clear recommendation for the women to go.” Interview 4, Line 466, Most important information is recommendation to screen

From the interviews it became clear that most breast cancer screening programmes sent a DA, alongside an invitation for a scheduled appointment. This seems at odds with wanting the recipient to read all the information about screening *before* making a decision about whether they want to undergo screening. Shifting the responsibility to an individual to cancel an existing appointment, might be seen to be a strategy to increase uptake at the cost of allowing an informed choice. Most participants did not give a reason for sending invitations with a scheduled appointment, though one explicitly stated that this was for the purpose of increasing attendance. None of the fetal trisomy anomaly screening interview participants described sending a scheduled appointment for screening with information, although their approach was dependent on the individual booking an appointment or informing their general practitioner (GP) about their pregnancy. (In the UK, GPs are HCPs who treat common medical conditions and make referrals to hospitals and other services for urgent and specialist medical treatment [33].) The time given to individuals to make a decision about whether to proceed with being screened varied, though in a few cases (mostly fetal trisomy anomaly), people were only given the length of the initial information-giving meeting to finalise their choice.

“The invitation comes with a pre-scheduled appointment.” Interview 11, Line 171, Invite with scheduled appointment

“I think that it’s generally done with the midwife sitting down, at the first meeting with the woman, which might be eight weeks, six weeks, and talking to the woman about this, and showing her pictures and information, and offering her the test.” Interview 3, Line 539, Decision by the end of appointment

“So, usually it’s [the time gap between receiving the information and making a decision to go to screening] at the same appointment. So, someone comes in, has a question or an offer is made, and the discussion is, occurs at that point and the referral is done at that, all at that same visit.” Interview 7, Line 441, Decision by the end of appointment

Most breast cancer participants described specific strategies for increasing uptake, including sending invitations to meetings to discuss screening with a scheduled appointment, or using “communicators” / health literacy experts on the content of DAs. They also described promoting screening through social media, or via patient letters from GPs, or HCPs “encouraging” women to attend screening, or financially incentivising HCPs for their screening uptake rate.

“When they, when the receive their first letter, they’re actually offered an appointment in the letter that we said if we [Audio breaks] say ‘Dear Miss Grant, we’ve made appointment with you at two o’clock next Thursday.’ If she looks at her diary and sees that that doesn’t suit, she’s sort of already engaged like she’s already imagining herself attending, and she’s more likely to ring up [...] whereas if we just say, ‘Oh, you’re eligible. Call this number and we’ll make an appointment,’ we get far fewer people joining up.” Interview 2, Line 295, Increasing uptake

“Some [regions], there is remuneration for the family physician if they achieve a percent target of eligible population that undergo screening. So, and it’s graded by the percentage of the target population that’s, achieved.” Interview 7, Line 196, Increasing uptake

“Family and friends, but also your family doctor so your GP [...] would be encouraging you to opt-in to the programme.” Interview 11, Line 297, Increasing uptake

#### Increasing informed choice or comprehensibility?

Our second theme involved the tension between improving informed choice or comprehensibility. The concept of informed choice requires that enough information is provided for someone to be able to fully understand all the options in their decision. Although, the concept also requires the information to be provided in a way that the reader can easily comprehend, i.e., without a large amount of medical terminology, or long and complex words or sentences. However, clear presentation almost always requires reducing the complexity, depth, and volume of information. This tension could be seen in our participants’ descriptions of the purpose of the DAs as well as in their descriptions of development of the content.

Some participants expressed the view that their DA prioritised informed choice i.e., more information / complexity over more comprehensibility, while others expressed the opposite view. (Only one participant expressed both types of comments.) Most participants expressing either kind of preference were from breast screening programmes, and almost all participants from the breast screening programmes expressed a preference. (In one country there was a clear legal requirement to provide information in a way that people understand).

“We’ve done research a few years ago to ask whether this information is maybe it’s too much […] But most women preferred the extended information.” Interview 13, Line 300, Informed choice over comprehensibility priority for DA

“I personally think we still, are… too much aiming at the more educated people [in the DA].” Interview 8, Line 777, Informed choice over comprehensibility priority for DA

“We try to make nothing more than two pages, so front and back. Like we wanted it to be a single, something easy. A lot of the ones we’ve used [in the region] before have been really quite long. Which, is not helpful. Nobody’s got time for that. [Laughs].” Interview 14, Line 35, Comprehensibility over informed choice priority for DA

When this potential tension between informed choice and comprehensibility was highlighted to participants, many felt that it could be addressed by the provision of multiple tiers, or levels, of information. Most countries provided two tiers. Typically, this was a more basic level provided through a DA such as a short and simply-worded leaflet, with a second, more extensive level of information through a website. On one occasion, however, the leaflet was more in depth than the website content (although there were also different “layers” of complexity online). Some interview participants suggested that this is a way that information can be provided in a more comprehensible way so that *everyone* can understand. While others requiring more, or more detailed information to feel fully informed, can access the secondary tier for more extensive information. These participants were almost always involved with a breast screening programme. One participant suggested a ‘cascade’ approach, where information was deliberately staggered, with smaller amounts of new information provided over time.

“We try and write everything to the level that most people can understand even with low literacy. So, we don’t have a differential… I mean, okay. No, maybe that’s not quite right. On our website, we would have additional information for people who really want to dig into the areas.” Interview 3, Line 898, Tiered information

“We think a lot about writing in [the country’s main language], easily understandable, words then having this [DA], you can go to this background information if you want to read more.” Interview 9, Line 903, Tiered information

#### Documentary analysis

**Fig. 1:**
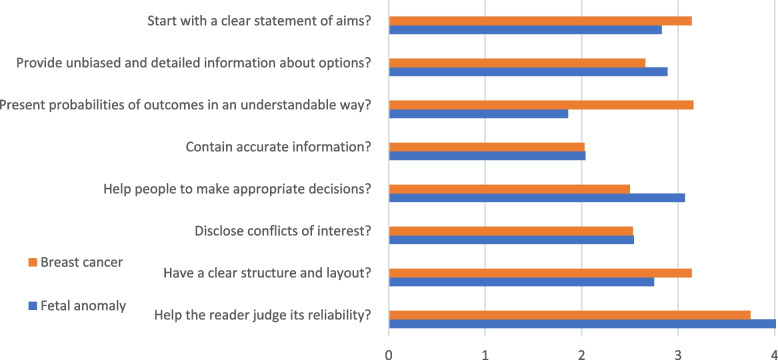
Average (mean) quality assessment scores for breast cancer and fetal trisomy anomaly screening discussion aids

In total, 15 DAs were assessed using the Picker Institute IPDAS checklist [13]. Each of the sub-domains for each of the eight domains was rated from one to five, with higher scores indicating that the DA more fully met the sub-domain statement, for example, “Describes its purpose” (see Additional file 2). The scores each researcher gave each sub-domain of a domain were averaged (mean) to reach a score for the domain, rounded to two decimal places. The means from both researchers were averaged to produce a mean for that domain for a specific DA. A mean was then calculated for all the fetal trisomy anomaly screening DAs for each domain, and likewise for all the breast cancer screening DAs (Fig. 1).

Overall, DAs for breast cancer scored higher, indicating better quality of information for making an informed choice, when compared to those for fetal trisomy anomaly screening (22.91 points vs. 22.04 points [highest possible score was 40]. The *Help the reader judge its reliability* domain scored highest for both conditions. The *Present probabilities of outcomes in an understandable way* domain showed the greatest difference in quality between the DAs for breast and fetal trisomy anomaly screening. Fetal trisomy anomaly screening DAs had comparable scores to breast cancer DAs in all domains, with the exceptions of this, and *Helping people to make appropriate decisions*, where fetal trisomy anomaly scored higher. Both scored low in *Contain accurate information, h*owever, this is not to say that the DAs provided *inaccurate* information; more that they tended not to provide information, or its source.

### Increasing informed choice or increasing uptake?

Interview results about the main purpose of the DAs, and the most important information, were supported in the documentary analysis. Benefits and harms associated with breast cancer screening were clearly described across countries. However, while all fetal trisomy anomaly screening DAs mentioned some form of benefits and harms, these were not always clearly described as such. Breast cancer DAs usually presented estimates of how common the condition was, though this was less common for fetal trisomy anomaly screening DAs. Sources of information were rarely cited, and personal opinions were not clearly distinguished from evidence-based information. Failing to provide accurate enough information to understand the prevalence of a condition is a barrier to allowing informed choice, as the likelihood / risk of the condition being screened for would not be fully understood.

Likewise, results from the documentary analysis revealed that DAs typically do not clarify what they will cover. However, the DAs were generally clear that the person had a decision to make, and that it was their own decision - “*It is your choice*”, which would aid informed choice. We found that many DAs simply stated what the condition was, without further definition or further detail. That lack of further definition seems likely to detract from people being fully informed. We consider that this, coupled with the lack of clarity on the benefits and harms of screening or of choosing not to be screened could result in increased uptake at the cost of informed choice.

DAs were also not clear in allowing people to consider priorities, motivations, and treatment outcomes that might affect their choice of action. For example, they lacked descriptions that might allow the reader to envisage living with breast cancer or with the effects of breast cancer treatment, or to have a child with an anomaly. For both breast cancer, and fetal trisomy anomaly screening DAs, the authors’ or developers’ credentials were clearly included but the source of funding to develop the DA was rarely reported, which hinders the reader’s ability to assess the reliability of the information provided, and consequently informed choice.

### Increasing informed choice or comprehensibility?

From the documentary analysis, we found that DAs typically do not clarify what they will cover, and so do not make it clear that the purpose of the material is to aid decision-making. This could potentially be to the detriment of both informed choice and comprehensibility, as if the reader does not understand what the DA will cover, or its purpose, then this is a barrier to being fully informed of their choices, or even realising that there a decision to be made. Likewise, not setting out the purpose or what the DA will cover may make the information more difficult to navigate, and / or understand. Both breast cancer and fetal trisomy anomaly screening DAs scored around three out of five for “having a clear structure and layout” (3.14 and 2.75 respectively). The DAs generally lacked clear sections, summary boxes or bullet points, suggesting that comprehensibility is not necessarily being prioritised.

Breast cancer DAs scored more highly for presenting probabilities of outcomes in an understandable way, using event rates, comparing outcome probabilities using the same numerator/denominator and using visual diagrams of outcome probabilities. DAs usually provided a general impression of how common the condition is, e.g., *“1 in 8 women in Australia will develop breast cancer in their lifetime*”, while this level of information was not consistently included in fetal trisomy anomaly screening DAs. From the materials that were assessed, fetal trisomy anomaly screening DAs rarely used visual diagrams, or gave outcome probabilities, beyond describing a chance as “high” or “low”.

In addition, fetal trisomy anomaly DAs rarely illustrated information with pictograms or pictures. If pictures appeared they were not always clearly labelled, further reducing comprehensibility. Without these visual means to assist understanding comprehensibility may be low, and people are less likely to understand their options thus decreasing the likelihood of informed choice.

Sources to allow people to find more information were available and provided in most DAs, although the sources of evidence on which statements within the DAs were made were rarely given. However, while for most readers this would likely confirm the credibility, or create more trust, it might also detract from comprehensibility.

In summary, we discovered that (i) there is a tension between informed choice and uptake and (ii) that attempts to achieve informed choice via decision aids generate tensions between improving comprehensibility and uptake. These tensions emerged in both the interviews and documentary analysis, and their impact can be observed, though this is not always consistent throughout all stages of development and distribution.

## Discussion

In this study we set out to investigate informed choice in screening programmes across eight countries. Achieving informed choice is difficult, even though it is often the espoused philosophy of screening programmes. In this paper, we have set out two tensions that exist in screening programmes, (1) the tension between increasing informed choice and increasing uptake; and (2) the tension between increasing informed choice and comprehensibility.

### The tension between increasing informed choice and increasing uptake

Although most countries have measures such as screening programmes in place for detecting conditions amongst asymptomatic people, uptake of screening provision can be variable and some countries may not have the resources available to increase their uptake. Across countries in Europe, for example, examination coverage for breast cancer screening (organised plus opportunistic), varies from 25 to 84.1% [34], and from 25% – <75% pregnant women use fetal anomaly screening (non-invasive pregnancy testing) [35].

We found that in DAs increasing informed choice can be seen as being at tension with maximising uptake. This perceived tension was highlighted by some of the participants in our study who articulated a concern that the way risk and benefit information is presented in DAs may deter some people from attending screening, or who explicitly stated that increasing informed choice was more of a priority than increasing uptake. While participants often reported that there was no set target for uptake, none suggested that there was a direct measure of informed choice for screening programmes. This approach seems to be paradoxical and may reflect a lack of clarity in the overarching objectives of screening programmes. A recommendation from our study would be to move toward recording levels of informed choice for screening.

Strech (2014) has highlighted that many countries record participation figures for screening (i.e., uptake) and this is often used as a de facto measure of informed choice (or effectiveness) for the programme, although this is directly at odds with the aim and ethos of many screening programmes. As Perkins and Repper (1999) warn, “to confuse informed choice and compliance is a mistake” (p.119). While fetal trisomy anomaly screening participants were more likely to report views that informed choice was more important than increased uptake, our documentary analysis determined that benefits and harms were less likely to be clearly presented in fetal trisomy anomaly DAs. Raffle (2001) makes the point that concerns about whether information presented in a DA may “put people off” (rather than its accuracy) reflects a confusion about whether the purpose of the DA is to increase uptake, or to increase informed choice. With clarity that informed choice is the purpose of a DA, a developer would not need to be so concerned about presenting the risks of screening, rather they might want to ensure that the consequences of the condition, the reader’s susceptibility, and the efficacy of screening were properly raised, so that the reader would understand the risks, as well as the effectiveness of screening. We would also recommend that organisations could hire specific personnel to oversee DA production though all stages of development and distribution, or create a refined checklist or tool for monitoring, to ensure that a programme’s overarching aim of either maximising uptake or increasing informed choice is reflected at every level.

### The tension between increasing informed choice and comprehensibility

A decision made to accept or decline a screening test should be based on access to accessible, accurate, evidence-based information [6]. Screening guidance acknowledges that there is a delicate balance between providing all the information that people invited to screening may need to make their decision, and avoiding “providing so much information that many are discouraged to read it” [6]. Balancing informed choice and comprehensibility may appear to be an irresolvable tension for screening decisions, as providing a DA with so much complex information that few can understand it (or will read it), or with so little information that it does not fully inform the reader are both counterproductive. However, we found that the provision of basic and then more detailed tiers of information appears to be the preferred option for our participant countries, and almost all DAs were seen by the participants as delivering a ‘basic’ level of comprehensible information. Typically, an additional level of further, more detailed information was made available from another source, such as a website.

Despite most of our participants’ organisations using some type of health literary expert, and / or a “lay members” panel to provide input on the content of their DAs to improve comprehensibility (further detail in our full report [36]), our documentary analysis found many DAs lacking in the domains that related to comprehensibility, such as *Present probabilities of outcomes in an understandable way*, which showed the greatest difference in quality between the DAs for breast and fetal trisomy anomaly screening.

The scores for these could easily have been improved by including pictures or visual aids, especially regarding probabilities of outcomes. However, there may be a lack of consensus around how comprehensibility may best be achieved. For example, one of the domains that the IPDAS checklist assesses is *Present probabilities of risks and potential outcomes in an understandable way*, and best practice is assumed to involve providing event rates, and using visual diagrams to aid comprehension of these probabilities [13]. However, Raffle (2001) suggests that “[i]f participants need to understand that screening may lead to investigation and treatment for a condition that would never have caused a problem, then this can be communicated. It does *not* require an accurate quantitative estimate of likelihood in order to convey this” (p.95, emphasis added).

Overall, we found that DAs for breast cancer provided better display of information, especially around descriptions and quantifications of the benefits and harms than those of fetal trisomy anomaly screening. This difference may reflect the differences in the conditions, while earlier diagnoses, prognoses, and survival rates, may appear to be complex information for most, conveying this information may be simpler than advising a parent whether they wish to continue a pregnancy for a child with a life-threatening condition. As one participant stated, “Every time you, you talk about something where, a potential end point choice is termination of pregnancy, it is going to be controversial” (Interview 14).

### Implications for practice

Using DAs to achieve informed choice generates tensions between improving informed choice and uptake, and informed choice and comprehensibility. The aim of comprehensibility is fundamentally at tension with an aim of being fully informed of all the available information, in all its complexity, and this tension was described at many stages of development of the DAs during our interviews and documentary analysis. However, the problem of whether to tailor a DA aimed towards higher levels of informed choice or comprehensibility appears to have been overcome by providing (at least) two levels, which adequately cater towards both those that require less detailed, more basic information to feel fully informed, and those that require further, and more detailed information. A basic and then more detailed tiering of information appears to be the preferred solution to balancing the contradictory aims of improving informed choice whilst maintaining comprehensibility.

In addition, while those delivering screening programmes and creating and distributing DAs appeared to align to the ethos of informed choice over increased uptake, we found a lack of integrated thinking at all stages of the development and distribution of the DA, which could lead to the promotion of uptake at the cost of informed choice. Addressing potential tensions between informed choice and uptake, and informed choice and comprehensibility, must therefore be considered at all of these stages. The quality assessment checklists such as the IPDAS checklist [13], could be highly useful to screening programmes during the development stage.

### Strengths and limitations

One of the strengths of our study is its international scope. We were able to gather a range of views across eight different countries. We were unable to interview a participant from Australia on fetal trisomy anomaly screening, and it was also not possible to source fetal trisomy anomaly screening material for Australia. In addition, the person interviewed for New Zealand was the same for both breast and fetal screening, and both were covered in one interview. These variations may potentially have slightly reduced the diversity of experience and views reported. Further research may be needed to explore the idiosyncrasies of each of these countries in greater depth.

Our research only considered two screening programmes, which may reduce our findings’ transferability, i.e., “whether or not the findings apply or ‘fit’ in similar settings” [37] (p.127). We also would not expect our findings to necessarily be transferable to healthcare systems from Low- or Lower-Middle Income Countries [38].

A mixed methods approach allowed us to compare data from different sources, and to explore patterns and differences across different variables [39]. Data from both sources were gathered separately, though concurrently, with conclusions enriched as they were based on all data [39]. Interview data were collected by trained researchers following a semi-structured interview guide to ensure consistency, and allow comparison of responses across interviews. Qualitative codes were initially reviewed independently by two researchers to inform a coding framework and ensure consistency of coding. Transcripts were not returned to participants for comment or correction, though we sought feedback of our initial findings from our participants in the consensus principles workshop to enable us to further refine our analysis. A validated tool was used in the DA quality assessment, which was conducted independently by two reviewers. However, we did not explore how the format and content of DAs impact the interpretation of the information they provide, or how they are received and perceived by patients and the public. This is an important area of consideration for further research.

## Conclusion

Following data collection and analysis across eight countries we suggest that attempts by screening programmes to achieve informed choice via decision aids generate tensions between improving informed choice and uptake, and informed choice and comprehensibility. These tensions were found in both our interviews and documentary analysis. While informed choice appears to be prioritised over uptake, uptake can become prioritised at the cost of informed choice during various points in delivery and distribution; perhaps due to lack of integrated planning. This problem may be due to a lack of clarity over the underlying aims and values of screening programmes.

This lack of clarity over the underlying aims and values of screening programmes will need addressing as it inevitably leads to discrepancies and tensions in how, when and where information is provided to those invited for screening. To counter this, organisations providing screening need to decide whether their overarching aim is maximising uptake or increasing informed choice, and once decided, this aim should be reflected in every level, and all information materials, including DAs. Organisations could hire specific personnel to oversee DA production though all stages of development and distribution of the DAs, or create a refined checklist or tool that covers points for monitoring where the method/s of invitation or distribution may result in promoting uptake at the cost of informed choice.

However, our findings also suggest that comprehensibility of the DA must be considered. Prioritising informed choice as an aim may lead to disregarding of comprehensibility, by providing too much information or complexity in an effort to ensure that the reader feels fully informed. However, none of our participant countries considered the DA alone to cover both those requiring a smaller amount of basic information to feel informed, or those that required more, and more complex information. The DA was one part of their provision of information. Typically, DAs were designed to provide a ‘basic’ and comprehensible tier of information, with at least one more detailed tier of information, to address the needs of both of these types. However, there are many methods to increase understanding of complex health messages available and we recommend that screening programmes take full advantage of developments in this area to improve the comprehensibility of their screening information to ensure that they are appropriate for all sectors of their populations.

## Supplementary Information

**Additional file 1.** Interview schedule used for data collection.

**Additional file 2.** Screenshot of report page showing revised IPDAS checklist used for documentary analysis.  

## Data Availability

The data that support the findings of this study are available from National Screening Committee but restrictions apply to the availability of these data, which were used under license for the current study, and so are not publicly available. Data are however available from the authors upon reasonable request and with permission of National Screening Committee.

## Abbreviations

DA: Decision Aid
GP: General Practitioner
HCP: Healthcare Professional
IPDAS: International Patient Decision Aid Standards Collaboration
NSC: National Screening Committee
