# Supplementary material for: Investigating informed choice in screening programmes: a mixed methods analysis
Source: BMC Public Health. 2022 Dec 12;22:2319. doi: 10.1186/s12889-022-14685-6 (PMC9743591; doi:10.1186/s12889-022-14685-6)
Supplement: Supplementary file 1 — Additional file 1. Interview schedule used for data collection. [file 12889_2022_14685_MOESM1_ESM.docx]

File name: Additional file 1

File format: Word document, docx

Title of data: Interview schedule

Description of data: Interview schedule used for data collection.

**Thank you for taking the time to speak to us:** Your expert inputs and perspectives will be extremely valuable to help us understand the choices people make about breast screening/early pregnancy fetal anomaly screening in your country. There are no right or wrong answers, and the discussion aims to allow us to better understand the overall structure and information provision in the screening service.

**Just to confirm:**

- Your participation is voluntary, and you are free to stop our conversation at any time without having to give a reason for doing so.
- This is expected to last about 40 minutes.
- We will record the conversation with your permission. This will help us transcribe and summarise the information, but we will not include any details that might identify you in any reports or publications.
- This summary findings from our discussion will be reviewed by researchers involved in the project (from Warwick University and Birmingham University).
- Do you give consent to have a conversation with me?
- Do you have any conflict of interest to disclose?

**For fetal trisomy anomaly- just before I start,** I would like to clarify that we are specifically interested in early pregnancy screening (10-14 weeks; Down's Syndrome, Edwards' Syndrome and Patau’s Syndrome), and not the late-stage structural anomaly screening.

Essential items highlighted.

|  | Questions | Comments | Tick if Asked |
| --- | --- | --- | --- |
| **1. I would like to start with some opening questions to learn more about your organisation and your role in relation to screening service in your country.** | | | |
| 1.1 | Is your organisation responsible for the development of information about screening?  ***Internal check:***  *i) Organisation type (equivalent to PHE/NSC)*  *ii)Make decisions (yes/no)?*  *iii)Run program (yes/no)?*  If not i, ii, or iii, thank and close interview, and ask for referral. |  |  |
| 1.2 | Could you briefly describe your role and responsibilities towards the screening program?  ***Internal check:***  *i) National*  *ii) Local or Provincial- Geographical regions*  *iii) Others (Site Practitioner- Midwives, Screening coordinator)* |  |  |
| 1.3 | How long have you worked in this role? |  |  |
| 1.4 | What are the topic areas that your role covers? |  |  |
| **2. Could you please talk me through the overall structure of the screening service in your country/ region** | | | |
| 2.1 | How is the screening organised?  Prompts:   - *What organisations are involved in the screening?* - *Do you use population call-recall (for breast-cancer screening) / opportunistic screening*? - *What are the eligibility criterion for population call/recall (for breast cancer screening)?* - *Are these decisions National/ regional/ local?* |  |  |
| 2.2 | How are people invited for the screening?  Prompts:   - *How is it managed?* - *What is the mode of invitation? For example, text messages, letters?* - *Usually, who contributes to decision making around the time of the invitation? What is the standard process? (For example, is there typically midwife involvement?)* |  |  |
| 2.3 | Is there a payment scheme for attendance at screening?  Prompts:   - *Are people incentivised/paid to attend?* - *Are GPs/heath care provider incentivised? (be sure to clarify if the payment is for invitation or attendance)* - *Do people have to pay to get screened?* |  |  |
| 3. **Now I would like to discuss about the approaches towards supporting informed choice in your screening service** | | | |
| 3.1 | What do you do to support people in their choice whether to attend the breast screening or fetal anomaly screening? |  |  |
| 3.2 | What form of material do you use to provide information about breast screening (or fetal anomaly) screening? *For example, text, leaflets, websites, national campaigns, anything else?*  Prompts:   - *Alongside / not alongside screening invitation?* - *Mode of delivery. For example, postal, email etc.* - *Who (what body) sends the invitation?* - *What is the overall length of information? (Multiple page booklet, single page leaflet)* - *What are the time gaps from when people receive the materials and their need to make a choice to screen?* - *In UK we call the information materials as ‘Screening information leaflet/booklet’. What do you refer them in your service/country?* |  |  |
| 3.3 | What are the main aims of the materials?  Prompts:   - *Out of these aims (re-state aims), what two aims are the priority for communication? As examples, understanding of the test, testing process, clarifying the benefits, explaining risk/possible harm* - *How are these priorities decided upon?* - *How do you make decisions about discussing the benefits versus harms, explaining follow on treatment options and choices ?* - *How do you make decisions about whether to promote uptake or informed choice?* - *How do you balance the length and amount of information provided with accessibility?*   **dig deep with different wordings to identify the priorities from the organisational point of view and possibly participant’s personal point of view.*  *For informed choice, what are the most important pieces information to convey? And why – what process did they take to decide if these were the priority?* |  |  |
| 3.4 | Who produces the content of the material-what body?  Prompts:  *Focus on who signs off final content? (Who agrees that?)* |  |  |
| 3.5 | How is the design of screening information in the materials organised at the program-level?  Should include:   - *How do you decide what goes into the information?* - *How is the information agreed upon?* - *Balance of experts versus user input and expert input (for example, ethicist)?* - *Is behavioural science considered?* - *Do they use an ethical framework?* - *Are there any evaluations carried out?* - *Is there public consultation?* - *Underlying framework/ guidance? Do you have SOPs or protocols, standard protocol for developing information?* - *Are the guidelines published?*   *If ‘No’-Are there any unpublished guideline/ guideline published under certain condition that you would like to share? What are those condition?* |  |  |
| 3.6 | To what extent is the information in the materials mandated centrally (a ‘standard version’), and is there any scope for local customization?  3.6 a) and if so, whether local customisations are agreed in any way?  Prompts:   - *Customization of material, different screening options, focus on material.* - *Can content deviate from the final version?* |  |  |
| 3.7 | Are there any alternate versions, such as those to improve equality, diversity, inclusion?  Prompts:   - *Whether it is adapted, translated or simplified e.g ‘easy reads, animations, pictorial?* - *What groups are considered when making the information accessible – such as age, gender, disability, or culture? What dimensions are important?* - *How is the information tailored to be better accessible to these groups?* - *What different formats are they available in?* - *How do you address the trade-off between* *inequality and uptake between different groups? More information might only reach the most educated and lead to inequalities* - *What processes are in place to understand the level of literacy required to understand the information?* (Any *automated tools to look at the literacy level?)* - *What literacy level is needed?* |  |  |
| 3.8 | *A*re the materials updated regularly?  Prompts:   - *What are the intervals (how frequently)?* |  |  |
| 3.9 | Are there any other environments (Such as ‘early bird’ pre-natal classes or counselling sessions) where the part of information is usually delivered?  *If yes, how?* |  |  |
| 3.10 | Overall, are there any controversies and challenges around the information materials?  Prompts:   - *How do you deal with these controversies/ challenges?* |  |  |
| **4. Before we conclude, just a few final points around legality** | | | |
| 4.1 | What, if any, information is legally required on the information material?   - *Which ones are of top priority?* - Are there any other legal obligations relating to informed choice for breast screening/fetal anomaly screening in your region/country? |  |  |
| Last but not least, do you have any question for me? | | | |
