# Supplementary material for: Investigating informed choice in screening programmes: a mixed methods analysis
Source: BMC Public Health. 2022 Dec 12;22:2319. doi: 10.1186/s12889-022-14685-6 (PMC9743591; doi:10.1186/s12889-022-14685-6)
Supplement: Supplementary file 2 — Additional file 2. Screenshot of report page showing revised IPDAS checklist used for documentary analysis. [file 12889_2022_14685_MOESM2_ESM.docx]

File name: Additional file 2

File format: Image in Word document, docx

Title of data: Table 7: Revised IPDAS checklist, section I (content)

Description of data: Screenshot of report page showing revised IPDAS checklist used for documentary analysis.


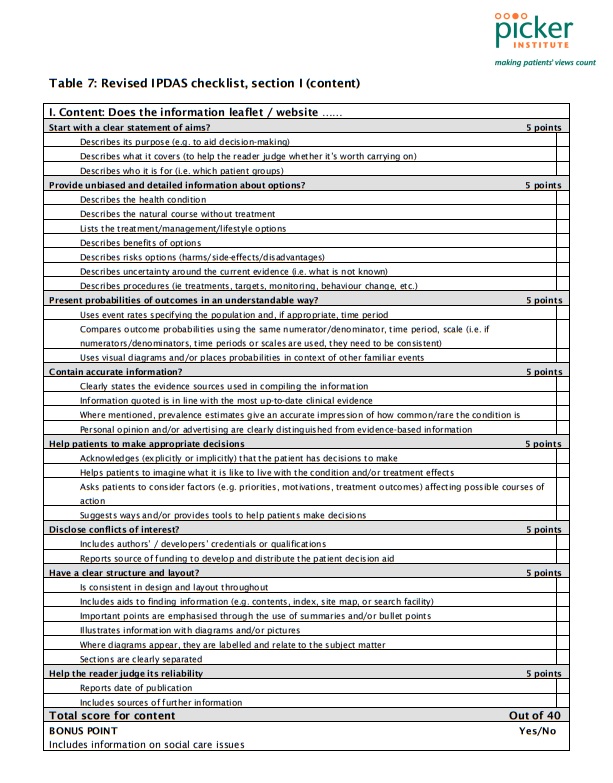


From: Coulter A, E.J., Swain D, Clarke A, Heron P, Farhat R, et al., *Assessing the quality of information to support people in making decisions about their health and healthcare* 2006, Picker Institute Europe: Oxford. (p.74).
